# Do changes in *Lactuca sativa* metabolic performance, induced by mycorrhizal symbionts and leaf UV-B irradiation, play a role towards tolerance to a polyphagous insect pest?

**DOI:** 10.1007/s11356-023-26218-8

**Published:** 2023-03-14

**Authors:** Marco Santin, Valeria Zeni, Arianna Grassi, Renato Ricciardi, Ylenia Pieracci, Filippo Di Giovanni, Sofia Panzani, Christian Frasconi, Monica Agnolucci, Luciano Avio, Alessandra Turrini, Manuela Giovannetti, Monica Ruffini Castiglione, Annamaria Ranieri, Angelo Canale, Andrea Lucchi, Evgenios Agathokleous, Giovanni Benelli

**Affiliations:** 1 Department of Agriculture, Food and Environment, University of Pisa, Via del Borghetto 80, 56124 Pisa, Italy; 2 Department of Pharmacy, University of Pisa, Via Bonanno 6, 56126 Pisa, Italy; 3 Department of Life Sciences, University of Siena, Via Aldo Moro 2, Siena, Italy; 4 Interdepartmental Research Center Nutrafood—Nutraceuticals and Food for Health, University of Pisa, 56124 Pisa, Italy; 5 Department of Biology, University of Pisa, Via L. Ghini 13, 56126 Pisa, Italy; 6 Department of Ecology, School of Applied Meteorology, Science & Technology (NUIST), Nanjing University of Information, Nanjing, 210044 China

**Keywords:** Anthropogenic change, Chemical ecology, Priming, *Spodoptera littoralis*, Noctuidae, Plant nutraceutical, Feeding behavior, Plant–insect tolerance

## Abstract

The increased ultraviolet radiation (UV) due to the altered stratospheric ozone leads to multiple plant physiological and biochemical adaptations, likely affecting their interaction with other organisms, such as pests and pathogens. Arbuscular mycorrhizal fungi (AMF) and UV-B treatment can be used as eco-friendly techniques to protect crops from pests by activating plant mechanisms of resistance. In this study, we investigated plant (*Lactuca sativa*) response to UV-B exposure and *Funneliformis mosseae* (IMA1) inoculation as well as the role of a major insect pest, *Spodoptera littoralis*. Lettuce plants exposed to UV-B were heavier and taller than non-irradiated ones. A considerable enrichment in phenolic, flavonoid, anthocyanin, and carotenoid contents and antioxidant capacity, along with redder and more homogenous leaf color, were also observed in UV-B-treated but not in AMF-inoculated plants. Biometric and biochemical data did not differ between AMF and non-AMF plants. AMF-inoculated plants showed hyphae, arbuscules, vesicles, and spores in their roots. AMF colonization levels were not affected by UV-B irradiation. No changes in *S. littoralis*-feeding behavior towards treated and untreated plants were observed, suggesting the ability of this generalist herbivore to overcome the plant chemical defenses boosted by UV-B exposure. The results of this multi-factorial study shed light on how polyphagous insect pests can cope with multiple plant physiological and biochemical adaptations following biotic and abiotic preconditioning.

## Introduction

Insect-plant interactions are routed by a hierarchy of physical and chemical cues, and their full understanding represents a fascinating ecological challenge (Braga and Janz 2021). For instance, host plants can induce chemical and morphological responses to face insect attacks (Sharma et al. 2021). Although this appears to be a bi-directional relationship, numerous abiotic and biotic factors can mediate it, further complicating the interactions (Sharma et al. 2021). Many microorganisms (e.g., arbuscular mycorrhizal fungi, AMF) can stimulate plant growth by facilitating the absorption of nutrients, enhancing the efficient use of soil natural resources, and promoting plant resistance to biotic and abiotic factors (Smith and Read 2008). In addition, AMF can alter plant secondary metabolism, leading to higher synthesis of antioxidant metabolites (Agnolucci et al. 2020); as such, AMF are increasingly considered a biotechnological tool for the sustainable production of safe and healthy plant foods, especially horticultural crops (Zhu et al. 2022; Messa & Savioli 2021; Fusco et al. 2022). AMF-related effects on the plant are commonly beneficial for both the insect and the plant itself (Sharma et al. 2017; Yu et al. 2022). Plant quality improves as a food source for insects when plant nutrient content is increased (Vannette and Hunter 2011). On the other hand, AMF may induce plant resistance by priming the jasmonic acid-dependent plant responses to phytophagous insects and changing the concentration and composition of terpenoids (Barber et al. 2013; Sharma et al. 2017). Such modifications may alter the plant attractiveness to insects, as well as insect behavior (Agathokleous et al. 2017; Masui et al. 2021; Sharma et al. 2017).

High intensity UV radiation, in particular the UV-B component (280–315 nm), can have a strong impact on several morphophysiological, molecular, and biochemical traits of plants (Jaiswal et al. 2022; Pandey et al. 2022a, 2022b; Rai and Agrawal 2021, 2022). Therefore, plants have evolved a fine UV-B perception mechanism and transduction pathway (Kliebenstein et al. 2002; Rizzini et al. 2011) to avoid intracellular impairments. Such responses lead to the increased content of reactive oxygen species (ROS)-scavenging and UV-B absorbing compounds, such as phenolic compounds (Brown et al. 2005; Favory et al. 2009; Santin et al. 2019, 2021a; Takshak and Agrawal 2019; Volkova et al. 2022). In addition, several studies reported a UV-B-triggered modulation in the content of photosynthetic pigments, such as chlorophylls and carotenoids (Carletti et al. 2003; Jansen et al. 2008; Santin et al. 2018, 2021b; Schreiner et al. 2012). These mechanisms are now understood to occur within the context of hormesis, where mild sub-toxic stress driven by a mild elevation of ROS activate signaling pathways and initiate adaptive responses that allow plants to cope with and prevent further harmful stress (Agathokleous 2021; Erofeeva 2022; Moustakas et al. 2022; Volkova et al. 2022). Due to the strong health-promoting properties of the bioactive compounds enhanced by the UV-B exposure, UV-B radiation has gained great attention as a green technology to improve the nutraceutical quality of agricultural plants in the last decades (Neugart and Schreiner 2018; Schreiner et al. 2012). Application of UV-B has been observed to have a positive effect on, e.g., basil (Mosadegh et al. 2018; Nascimento et al. 2020), rice (Faseela and Puthur 2018), chili pepper (Dolzhenko et al. 2010), mung bean (Wang et al. 2017), and wheat (Chen et al. 2019). Besides, UV-B radiation impacts insect-plant interactions, directly by affecting herbivore behavior or indirectly by altering plant biochemistry and morphology (Bornman et al. 2019; Prieto-Ruiz et al. 2019). In addition, the use of light-emitting diode (LED) illumination in the horticultural field is constantly expanding. Due to their energy efficiency, low radiant heat and durability as well as the possibility of customization in terms of wavelength emission and spectral composition, LED light represents an eco-friendly and economically sustainable solution as artificial lighting source (Bourget 2008; Bantis et al. 2018).

In addition, the secondary metabolites are major drivers of plant–insect herbivore interactions too, and the effects of such hormetic priming on plant–insect interactions are poorly understood even though UV priming is among the most promising priming approaches (Christou et al. 2022). Recently, UV-B radiation has also evolved as an environment friendly technology, with the potential of improving crop protection against agricultural insect pests, mainly by boosting both constitutive and inducible plant defenses (Escobar-Bravo et al. 2021; Qi et al. 2018). Studies on the influence of UV-B radiation on the production of volatile compounds are quite scarce (Jaiswal and Agrawal 2021; Johnson et al. 1999). Moreover, the use of UV-B LED light for horticultural purposes is at its infancy, with most current studies involving the use of UV-B fluorescent tubes for UV-B treatments. However, LEDs represent a valuable option, compared to UV-B fluorescent tubes, considering their longer lifespan, the higher energy efficiency, the negligible heat loss, and the higher customizability in terms of power and wavelength.

It is urgently necessary to reduce pesticide use in agricultural settings to avoid adverse effects on human health and the environment, as well as the rapid emergence of resistance in targeted species (Pavela and Benelli 2016). As a result, developing novel and eco-friendly pest management tools through habitat manipulation is a worthwhile research endeavor. Previous research has shown that both plant UV-B light exposure and mycorrhizal symbiosis can influence the arthropod feeding activity (Barber et al. 2013; Qi et al. 2018). However, little has been done to shed light on the potential effects of the interaction between UV-B radiation and AMF colonization of horticultural crops and their key arthropod pests (Zeni et al. 2023). In this framework, one may question whether UV radiation exposure and mycorrhizal symbionts can boost plant tolerance to polyphagous insect pests attacking horticultural crops. Therefore, the present study aims at unraveling the role of the combination of plant above- and below ground treatments (UV-B exposure and mycorrhization) in the feeding activity of key arthropod pests. To this end, we evaluated biochemistry, morphology, and physiology of *Lactuca sativa* L. plants with or without AMF and exposed or not to UV-B radiation as well as the feeding behavior of larvae of *Spodoptera littoralis* (Boisduval) (Lepidoptera: Noctuidae), a highly polyphagous insect that attacks over 40 plant families. We hypothesized that UV-B priming and AMF inoculation could enhance leaf defense potential and improve the performance of plants under herbivory.

## Materials and methods

### Insects

The insects tested here were mass-reared at the Entomology Lab of the Department of Agriculture, Food and Environment (DAFE), University of Pisa (Italy), under controlled conditions [27 ± 1 °C, 75% R.H., and 16:8 (L:D)-h photoperiod]. Batches of *S. littoralis* eggs were placed on filter paper in a plastic container. Newly hatched larvae were gently transferred on a semi-synthetic bean-based (Sorour et al. 2011). The larval development on semi-synthetic diet takes 18–20 days and includes 6 larval stages. In our feeding bioassay, we used 3^rd^–4^th^ instar larvae.

### Plant and fungal material

Organic seeds of the red leaf lettuce (*L. sativa* L. var. *crispa*) cv. Red Salad Bowl were bought from Landen company (Blumen Group, Milan, Italy). The research was carried out at DAFE, University of Pisa. The seeds were sterilized in a 5% sodium hypochlorite solution for 15 min with magnetic stirring, and then rinsed thoroughly with distilled water. The seeds were then sown on moistened filter paper in plastic trays (25 × 40 cm, 2 seeds cm^−2^). The trays were placed in climate-controlled chambers and kept in the dark at 24 °C for 72 h, followed by 16:8 (L:D)-h photoperiod. Blue/red (1:2 ratio) and green (10%) LEDs (C-LED, Imola, Italy) provided photosynthetic active radiation (PAR), with a photosynthetic photon flux density (PPFD) of 225 ± 5 μmol m^−2^ s^−1^.

The AMF species *Funneliformis mosseae* (T.H. Nicolson & Gerd.) C. Walker & A. Schüßler, isolate IMA1, was used in the experiment. The AMF isolate was obtained from pot cultures in the DAFE Microbiology Laboratory’s collection. The fungal inoculum was grown in a greenhouse for 6 months on *Trifolium alexandrinum* L. as a host plant in a mixture (1:1 by volume) of sterilized soil and calcined clay (OILDRI Chicago, IL, USA). At harvest, roots were cut into approximately 1-cm fragments and mixed with the substrate to form a homogeneous crude inoculum mixture, which was then air-dried and stored until use. Prior to the experiment, the biological activity of the inoculum was assessed using the mycorrhizal inoculum potential (MIP) bioassay described in Njeru et al. (2014), and a level of 50–60% was considered optimal.

### Mycorrhizal and UV-B treatments

Once the cotyledons were fully expanded (about 5 days after sowing), the sprouts were transferred to polystyrene plug trays (16 mL per cell, one sprout per cell) with a sterilized calcined clay as substrate. The substrate of half cells (120) was mixed (1:1) with *F. mosseae* IMA1 crude inoculum. To ensure a common AMF-associated microbiota to uninoculated control plants, the other half of the cells (120) received the same amount of sterilized crude inoculum (mock inoculum), and each cell received 2 mL of a filtrate obtained by sieving a mixture of mycorrhizal inocula through a 50-µm pore diameter sieve and a Whatman paper no. 1 (Whatman International Ltd, Maidstone, Kent (Koide and Li 1989).

The seedlings were irrigated twice a week with half-strength Hoagland’s nutrient solution (pH 6, 1.15 mS cm^–1^ electrical conductivity (EC)). Furthermore, plantlets were irrigated with distilled water (10 mL per pot) as needed. After 1 week in the growth chamber, mycorrhizal (+ M) and non-mycorrhizal (− M) plantlets were exposed or not to supplemental UV-B radiation for 2 weeks. UV-B radiation was provided by UV-B LEDs (High Power UV-B LTPL-G35UVB308GH, LITE-ON Technology, Inc., Taipei City, Taiwan) assembled by C-LED company (C-LED, Imola, Italy). The emission peak of the LEDs was 308 nm (half band width, 15 nm). The output power per LED was 62 mW, and the view angle was 120°. The UV-B irradiance at the top of the plants was 0.4 W m^–2^, and the treatment lasted 16 h per day (equivalent to a daily UV-B dose of 23 kJ m^–2^). Irradiance was measured using the spectrometer (FLAME-T-XR1-ES S/N: FLMT07829, Ocean Insight, Maybachstrasse 11, Ostfildern, D-73760 Germany) with fiber optics (QP400-1-UV-BX; Ocean Insight) and cosine corrector (CC-3-UV-S; Ocean Insight). During the treatment period and prior to sampling, both the + UV-B and − UV-B groups of plants were also exposed to PAR with a 16:8 (L:D)-h photoperiod, as indicated in the previous paragraph. Preliminary tests on lettuce plants were used to determine the UV-B dose. UV-B irradiation was carried out during the photoperiod’s 16-h light cycle, as previously described. To avoid the transfer of mycorrhizal fungi, the + M and − M plants in both the UV-B treatment and control chambers were placed in separate plastic trays. Two weeks after the start of the UV-B treatment (3 weeks after mycorrhizal inoculation), plants from all four groups (− M/ − UV-B; − M/ + UV-B; + M/ − UV-B; + M/ + UV-B; 60 plants per treatment) were randomly divided and sampled for further analysis.

### Biometric indexes

Five different plants (biological replicates) per experimental condition were harvested, and the total number of fully expanded leaves per plant (*n*) and plant height (cm) were determined. In addition, same plants were weighed to measure the fresh (FW; g) and dry weight (DW; 60 °C until constant weight). DW/FW ratio was also calculated and expressed as a percentage.

### Total phenolic, flavonoid, and anthocyanin extraction and determination

Total phenolics, flavonoids, and anthocyanins were measured in three separated groups of plants per treatment; each group consisted in five randomly selected freeze-dried plants (fifteen plants per group per treatment totally). Extraction was performed on 50 mg of freeze-dried sample using the method described by Tavarini et al. (2019) with few modifications. Briefly, samples were extracted with 1.5 mL of 80% methanol, and then sonicated for 30 min. After stirring for 30 min, the samples were centrifuged, and the supernatant was collected and stored at 4 °C. The pellet was subjected to a further extraction with 1 mL of 80% methanol, and the supernatants were combined and stored at 4 °C prior to the assays above.

The total phenolic content was determined using the Folin–Ciocalteau method (Borbalàn et al. 2003), with the absorbance at 750 nm through an Ultrospec 2100 pro-UV–vis spectrophotometer (Amersham Biosciences). The concentration of total phenolics was expressed as mg of gallic acid equivalents (GAE) g^−1^ FW.

Flavonoid concentration was measured, according to Kim et al. (2003), and the absorbance at 510 nm was recorded. The concentration of flavonoids was expressed as mg of catechin equivalents (CAE) g^−1^ FW. Commercial standards were used to create standard curves for total phenolic and flavonoid evaluation (Sigma-Aldrich Chemical Co., St. Louis, MO, USA).

Total anthocyanins were extracted and determined using the pH differential method described by Giusti and Wrolstad (2001). In brief, 50 mg freeze-dried samples were extracted in acidified methanol (1% HCl), and absorbance at 530 and 700 nm was measured. The following formula is used to calculate the final absorbance (*A*_f_) of the samples:

$${A}_{f}=\left({A}_{530}-{A}_{700}\right)\mathrm{pH}\;1.0-\left({A}_{530}-{A}_{700}\right)\mathrm{pH}\;4.5$$

Total anthocyanin concentration was expressed as µg of cyanidin-3-O-glucoside (molar extinction coefficient 26,900 L cm^−1^ mol^−1^; molecular weight 449.2 g mol^−1^) equivalents (C3GE).

### Total antioxidant activity evaluation

Total antioxidant activity was measured in freeze-dried phenolic extracts using the ABTS (2,2-azinobis (3-ethylbenzothiazoline-6-sulfonic acid) and the ferric reducing antioxidant power (FRAP) assays, as described by Re et al. (1999) and Benzie and Strain (1996), respectively. According to the ABTS assay, the absorbance was measured at 734 nm, and the antioxidant activity was expressed as μmol of Trolox equivalent antioxidant capacity (TEAC) g^−1^ FW. According to the FRAP assay, the absorbance was read at 593 nm, and the antioxidant activity was expressed as μmol of Fe (II) g^−1^ FW. Through the calculation of standard curves, the respective commercial standards (Trolox and FeSO_4_, considering the ABTS and FRAP assays, respectively; Sigma-Aldrich Chemical Co., St. Louis, MO, USA) were used in both antioxidant activity determinations.

### Chlorophyll and carotenoid determination

Chlorophylls *a* and* b* and total carotenoids were extracted and quantified spectrophotometrically (Ultrospec 2100 pro-UV–vis spectrophotometer, Amersham Biosciences) from three groups of five plants per group, following the method reported by Wellburn (1994) with few modifications. In brief, 150-mg samples were homogenized with 80% (w/v) cold acetone before being centrifuged (5500 × g per 5 min at 4 °C), and the supernatant was collected. A second volume of 80% cold acetone was added to the pellet, centrifuged (5500 × g per 5 min at 4 °C), and the resulting supernatant was combined with the first. This procedure was repeated until the supernatant after centrifugation was clear. The absorbance of the combined supernatant was read at 663, 648, and 470 nm.

### Color measurement

Color was measured on three different fully expanded leaves per plant, five plants per experimental condition, using a Konica Minolta CR-600 portable colorimeter (Holdings, Inc., Osaka, Japan). Color was determined using the CIELab system (CIE 1977), where L* (lightness), a* (redness), and b* (yellowness) were used to calculate chroma (C*; (a*^2^ + b*^2^)1/2) and hue angle (H*; tan^−1^ (b*/a*)) indexes (Priolo et al. 2000). Measurements were conducted in an 8-mm area diameter, specular component included, and 0% UV, D65 standard illuminant, observer angle 10°, and zero and white calibration.

### Analyses of AMF root colonization

For each treatment, the percentage of mycorrhizal colonization on the total root system of 12 plants was calculated. Following Phillips and Hayman (1970) method, each root system was rinsed with tap water, clarified, and dyed with 0.05% Trypan blue in lactic acid rather than phenol. The grid line intersection method was used to calculate the percentage of colonized roots length (Giovannetti and Mosse 1980). To check for the presence of intraradical AM fungal structures (e.g., appressoria and arbuscules), roots were mounted on slides with lactic acid and examined using a Reichert-Jung (Wien, Austria) Polyvar light microscope.

### Feeding assay with *Spodoptera littoralis* larvae

To determine the feeding preference of *S. littoralis* larvae towards different treated/untreated lettuce plants, a no-choice test was performed following the method of Vandenbussche et al. (2018) with slight modifications. A 3^rd^–4^th^ instar larva was isolated and gently transferred to a Petri dish containing a leaf of treated/untreated lettuce. To absorb the excess moisture released by the leaf, a filter paper was placed on the bottom of the Petri dish. The Petri dishes were stored in the dark to avoid the larvae visual orientation. The acceptability of the leaves to the larvae was evaluated by noting the proportion of consumed leaves after 18 h. A total of 40 leaves were used per treatment, with two leaves chosen at random for each plant. To evaluate the amount of consumed leaf, a picture of each leaf has been taken before the introduction into the Petri dish and after 18 h, using a Nikon D5300 digital camera (Tokyo, Japan). The leaves were placed on a square of white paper of a known area (10 cm × 10 cm). Each picture was cut out to the size of a square and converted to an 8-bit black-and-white image. ImageJ software was used to calculate the size of the leaf area (black area) in relation to the paper square (white area). Therefore, the percentage of the consumed leaf was assessed by comparing the initial leaf area to the remaining leaf area after 18 h of larval feeding.

### Statistical analysis

Differences in the biochemical parameters analyzed between the different groups of plants, considering both the mycorrhization (− M and + M) and the UV-B treatment (+ UV-B and − UV-B) were assessed with a two-way ANOVA followed by post hoc Tukey–Kramer test (*p* < 0.05) to evaluate significant interactions, and data are reported as mean ± standard error (SE). JMP software (JMP®, Version 16. SAS Institute Inc., Cary, NC, 1989–2021) was used to perform both the two-way ANOVA and a multivariate statistic with an unsupervised approach, a principal component analysis (PCA), and a two-way hierarchical clustering analysis (HCA). PCA combines all the *n* measured variables simultaneously (biometric, biochemical, and color parameters investigated in this study) to display possible clustering patterns according to newly generated *p* variables (*p* < *n*), the so-called principal components (PCs). In this study, the first two PCs, which explain most of the differences, were considered. The HCA (Euclidean distance, Ward’s linkage) was used to underline the similarity among the groups and the parameters considered. Data of mycorrhizal colonization were analyzed by independent Student’s *t*-test to assess the effect of UV-B treatment on inoculated plants, using SPSS statistical package v. 23.0. GraphPad Prism (v. 9.5.1, GraphPad Software, San Diego, California, USA) was used to analyse data collected from the no-choice assays on *S. littoralis. *The data were previously transformed with arcsine√ to evaluate their distribution. As the goodness-of-fit test evaluating the data distribution outlined that they were not normally distributed (Shapiro–Wilk test, *p* < 0.01), differences in larval acceptance of leaves were analyzed using the Kruskal–Wallis test. A probability level of *p* < 0.05 was used for the significance of differences between means.

## Results

### Mycorrhizal status of experimental plants

**Fig. 1:**
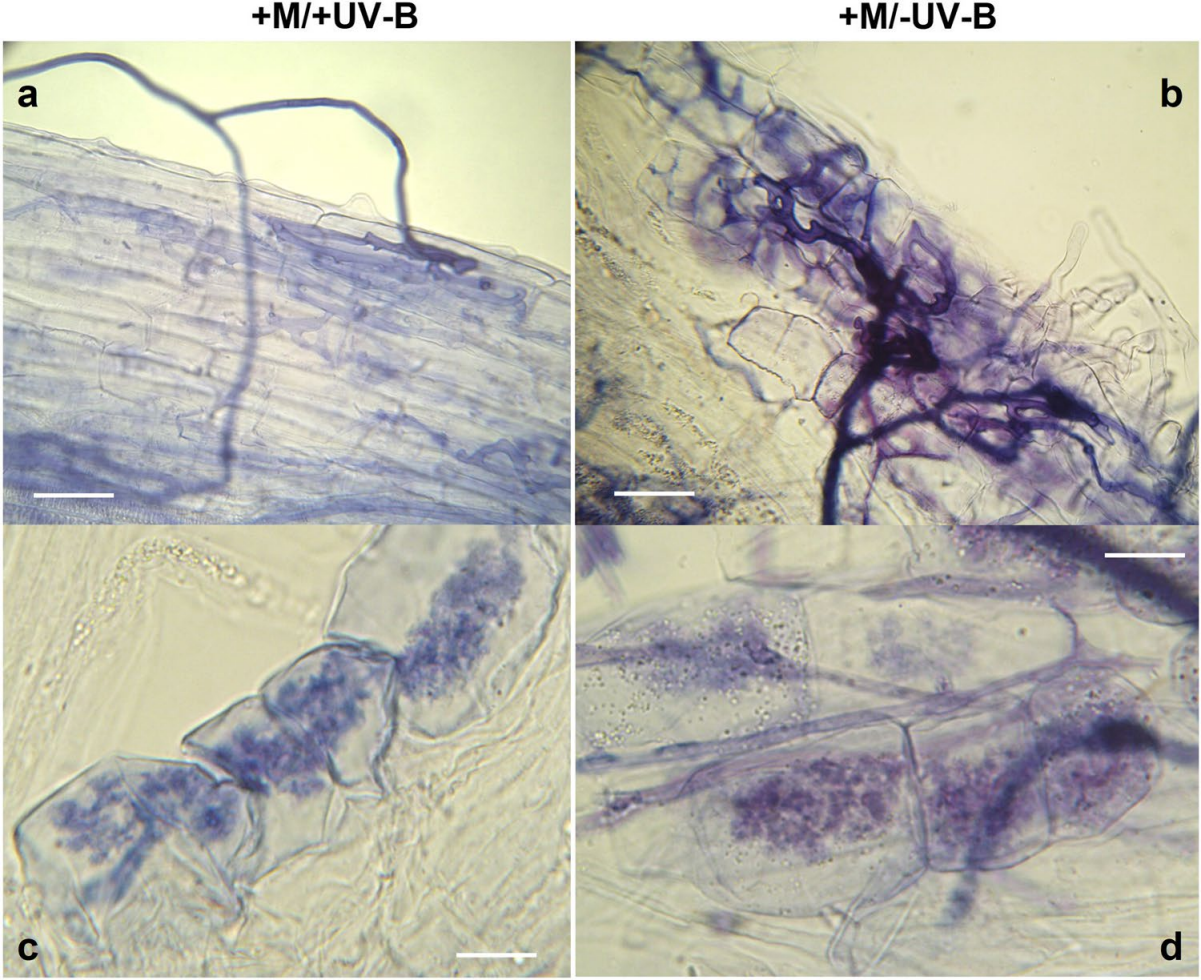
Light photomicrographs of fungal structures formed by *Funneliformis mosseae* IMA1 on the roots of *Lactuca sativa* L. (+ M) exposed or not to UV-B light (+ UV-B or − UV-B). **a**, **b**. Fungal extraradical and intraradical hyphae and appressoria formed on the root surface (scale bars: **a**, 30 µm; **b**, 20 µm); **c**, **d**. Arbuscules produced within cortical root cells (scale bar 15 µm)

Lettuce plants inoculated with *F. mosseae* IMA1, collected 21 days post-inoculation, independently of UV-B treatment, showed in their roots the typical symbiotic structures of AMF, such as hyphae, arbuscules, vesicles, and spores, detected under the optical microscope (Fig. 1).

On the contrary, control plants did not show any colonization, as expected, since they were mock inoculated. UV-B treatment did not significantly affect AMF colonization in inoculated plants (*t* = 1.66, *p* = 0.11). Mycorrhizal root length independent of UV-B treatment was 27.7 ± 9.3% (*mean* ± *SD*).

### Biometric indexes and dry matter content

Weight and length of the plants, number of leaves per plant, and dry matter content were evaluated (Table 1). The interaction between UV-B exposure and mycorrhization did not significantly affect any of the aforementioned traits. Considering only the mycorrhization, − M and + M plants did not show any significant difference in the parameters analyzed, except for the plant length; − M plants were on average 6.8% higher than the + M plants. UV-B radiation was effective in increasing both the plant weight and length (by 26.7% and 20.8%, respectively), while no significant differences were observed in terms of the number of leaves and dry matter percentage between − UV-B and + UV-B plants.

**Table 1:** Weight, length, number of fully-expanded leaves per lettuce plant, and dry matter content of mycorrhizal (+ M) or non-mycorrhizal (− M) lettuce plants, treated with UV-B radiation (+ UV-B) or not (− UV-B)

UV-B exposure	Mycorrhization	Weight (g)	Length (cm)	Leaves (*n*)	DW/FW (%)
− UV-B	− M	0.80 ± 0.08	4.32 ± 0.08	4.60 ± 0.19	10.08 ± 0.47
	+ M	0.92 ± 0.10	3.96 ± 0.05	4.20 ± 0.30	10.81 ± 0.97
+ UV-B	− M	1.13 ± 0.08	5.12 ± 0.08	4.80 ± 0.16	10.14 ± 0.28
	+ M	1.06 ± 0.07	4.88 ± 0.09	4.60 ± 0.19	9.67 ± 0.07
Mean effect					
− UV-B		0.86 ± 0.08 b	4.14 ± 0.08 b	4.40 ± 0.22	10.45 ± 0.51
+ UV-B		1.09 ± 0.06 a	5.00 ± 0.08 a	4.70 ± 0.15	9.90 ± 0.17
	− M	0.97 ± 0.09	4.72 ± 0.15 a	4.70 ± 0.15	10.11 ± 0.25
	+ M	0.99 ± 0.08	4.42 ± 0.17 b	4.40 ± 0.22	10.24 ± 0.50
ANOVA (*p*-values)					
Mycorrhization (A)		n.s	0.0065	n.s	n.s
UV-B exposure (B)		0.0352	< 0 .0001	n.s	n.s
A × B		n.s	n.s	n.s	n.s

The data show the *mean* ± *SE* (*n* = 5). Two-way ANOVA (*p* < 0.05) was used to determine statistically significant differences, and different letters indicate significantly different values according to the Tukey–Kramer test. Data related to the mean effects, as well as the respective statistics, are obtained by considering each factor (UV-B or mycorrhization) individually, merging the data corresponding to the other, not considered, factor

*n.s.* not significant

### Total phenolic, flavonoid, anthocyanin contents and antioxidant capacity

**Fig. 2:**
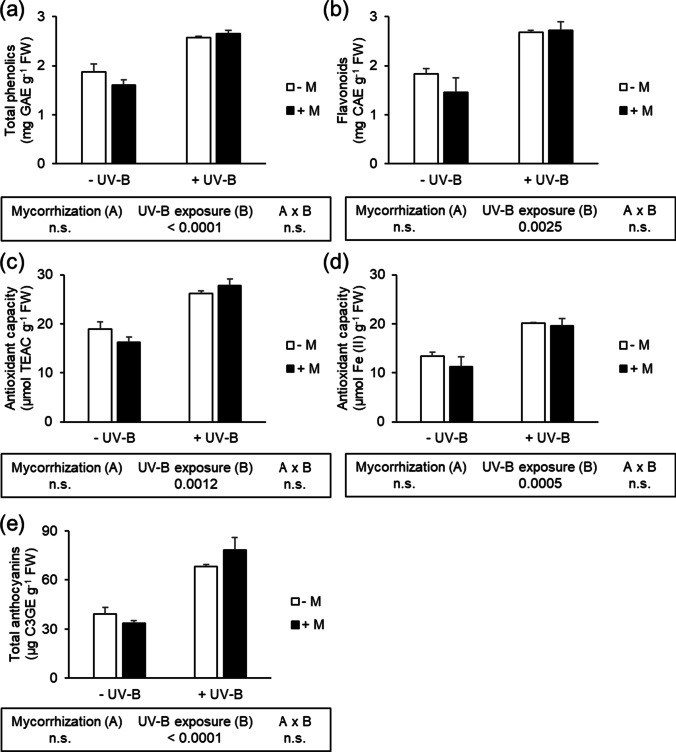
Determination of (**a**) total phenolics; (**b**) flavonoids; antioxidant capacity measured through (**c**) ABTS, and (**d**) FRAP assays; (**e**) total anthocyanins of mycorrhizal (+ M) or non-mycorrhizal (− M) lettuce plants, UV-B-treated (+ UV-B) or untreated (− UV-B). The two-way ANOVA results are shown in the box below each histogram. n.s., not significant. According to two-way ANOVA, there were no significant interaction effects (*n* = 3, *p* > 0.05)

The interaction between the mycorrhization and UV-B treatment did not produce statistically significant results in terms of total phenolics, flavonoids, antioxidant capacity, and total anthocyanins. Mycorrhizal and non-mycorrhizal lettuce plants did not significantly differ in total phenolics, flavonoids, and total anthocyanin contents, as well as in the antioxidant capacity measured with either ABTS or FRAP assay (Fig. 2). Conversely, UV-B exposure enhanced the content of these secondary metabolites. Specifically, lettuce plants exposed to UV-B radiation displayed a 53, 37, and 102% higher content of total phenolics, flavonoids, and anthocyanins, respectively. Similarly, the antioxidant activity of UV-B irradiated plants was significantly higher (28% in ABTS assay and 62% in FRAP assay), compared to the non-irradiated plants.

### Chlorophyll and carotenoid content

The interaction between UV-B exposure and mycorrhization did not significantly impact the chlorophylls *a*/*b* ratio, the sum of chlorophylls *a* + *b* or the total carotenoid concentrations (Table 2). No statistically differences were found between mycorrhizal and non-mycorrhizal plants in terms of all the photosynthetic pigments measured. However, UV-B radiation increased the concentration of total carotenoids by 45%, compared to the non-irradiated plants. No significant differences between UV-B irradiated and unirradiated plants were observed considering the chlorophylls *a*/*b* ratio and the sum of chlorophylls *a* + *b*.

**Table 2:** Chlorophyll *a*/*b*, chlorophyll *a* + *b*, and total carotenoid concentration of mycorrhizal (+ M) or non-mycorrhizal (− M) lettuce plants, treated with UV-B radiation (+ UV-B) or not (− UV-B)

UV-B exposure	Mycorrhization	Chl. *a*/*b* ratio	Chl. *a* + *b*	Carotenoids
			(µg g − ^1^ FW)	(µg g − ^1^ FW)
− UV-B	− M	1.73 ± 0.15	52.42 ± 7.92	4.90 ± 1.12
	+ M	1.47 ± 0.17	54.50 ± 7.20	5.25 ± 0.10
+ UV-B	− M	1.48 ± 0.12	55.50 ± 3.87	7.03 ± 0.69
	+ M	1.46 ± 0.07	47.22 ± 4.84	7.66 ± 0.86
Mean effect				
− UV-B		1.60 ± 0.12	53.46 ± 4.81	5.07 ± 0.51
+ UV-B		1.47 ± 0.06	51.36 ± 3.33	7.35 ± 0.51
	− M	1.60 ± 0.10	53.96 ± 4.00	5.96 ± 0.76
	+ M	1.47 ± 0.08	50.86 ± 4.21	6.46 ± 0.66
ANOVA (*p*-values)				
Mycorrhization (A)			n.s	n.s
UV-B exposure (B)			n.s	0.0204
A ˂ B			n.s	n.s

The data show the *mean* ± *SE* (*n* = 3). Two-way ANOVA (*p* < 0.05) was used to determine statistically significant differences

*n.s.* not significant

### Color determination

**Fig. 3:**
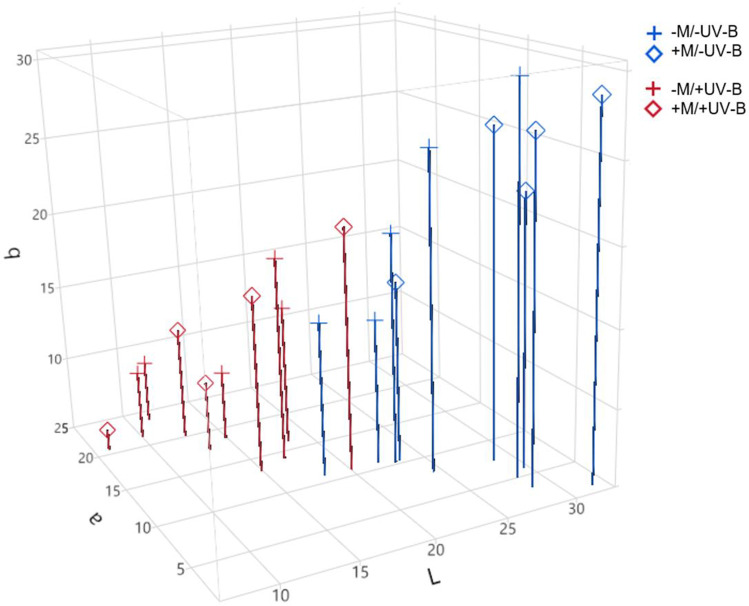
3D scatter chart setting L*, a*, and b* as coordinates, according to the CIE L*a*b* system. Different symbols refer to individual lettuce plants from the different groups referred to the mycorrhizal (+ M) or non-mycorrhizal (− M) lettuce plants, treated with UV-B radiation (+ UV-B) or not (− UV-B). Lightness (L*), redness (a*), and yellowness (b*) values for each plant are the mean of three independent measurements on three fully expanded leaves

UV-B exposure was the only factor significantly affecting the color of lettuce leaves (Table 3). Particularly, UV-B-treated plants showed 46, 44, and 45% smaller values of lightness (L*) and blue-yellow (b*) coordinates, and hue angle (H*), respectively, and 135% larger values of red-green (a*) coordinate. The mycorrhization and the interaction of mycorrhization and UV-B exposure were ineffective in significantly changing the aforementioned color parameters. To better visualize the clustering pattern according to the L*, a*, and b* coordinates, a 3D scatter chart was created (Fig. 3). The two main clusters detected in Fig. 3 corresponded to − UV-B (in the upper right portion of the space) and + UV-B (in the lower-left portion of the space) groups, regardless of the mycorrhization. Indeed, no clusters could be identified according to − M and + M plants, confirming that the mycorrhization did not modify the color parameters studied.

**Table 3:** Color parameters (L*, a*, b*, H*, and C*) in mycorrhizal (+ M) or non-mycorrhizal (− M) lettuce plants, treated with UV-B radiation (+ UV-B) or not (− UV-B). Lightness (L*), redness (a*), and yellowness (b*) were used to calculate chroma (C*; (a*2 + b*2)1/2) and hue angle (H*; tan^−1^ (b*/a*)) indexes

UV-B exposure	Mycorrhization	L*	a*	b*	H*	C*
− UV-B	− M	23.2 ± 1.7	9.0 ± 1.1	20.9 ± 3.0	60.8 ± 3.9	22.7 ± 1.6
	+ M	28.4 ± 1.4	5.9 ± 1.5	24.3 ± 2.1	70.6 ± 4.7	24.5 ± 1.6
+ UV-B	− M	14.4 ± 1.6	18.8 ± 1.7	12.2 ± 1.8	33.1 ± 6.0	23.1 ± 0.7
	+ M	13.6 ± 2.0	16.5 ± 1.9	13.2 ± 2.5	38.6 ± 8.1	22.4 ± 0.7
Mean effect						
− UV-B		25.8 ± 1.3 a	7.5 ± 1.0 b	22.6 ± 1.8 a	65.7 ± 3.3 a	23.6 ± 1.1
+ UV-B		14.0 ± 1.2 b	17.6 ± 1.3 a	12.7 ± 1.5 b	35.9 ± 4.8 b	22.7 ± 0.5
	− M	18.8 ± 1.8	13.9 ± 1.9	16.5 ± 2.2	47.0 ± 5.7	22.9 ± 0.8
	+ M	21.0 ± 2.7	11.2 ± 2.1	18.7 ± 2.4	54.6 ± 6.9	23.5 ± 0.9
ANOVA (*p*-values)						
Mycorrhization (A)		n.s	n.s	n.s	n.s	n.s
UV-B exposure (B)		< 0.0001	< 0.0001	0.0007	0.0001	n.s
A × B		n.s	n.s	n.s	n.s	n.s

Data represent the *mean* ± *SE* (*n* = 5). Statistically significant differences were evaluated by two-way ANOVA (*p* < 0.05), and different letters indicate significantly different values according to Tukey–Kramer test

*n.s.* not significant

### Feeding assay with *Spodoptera littoralis* larvae

**Fig. 4:**
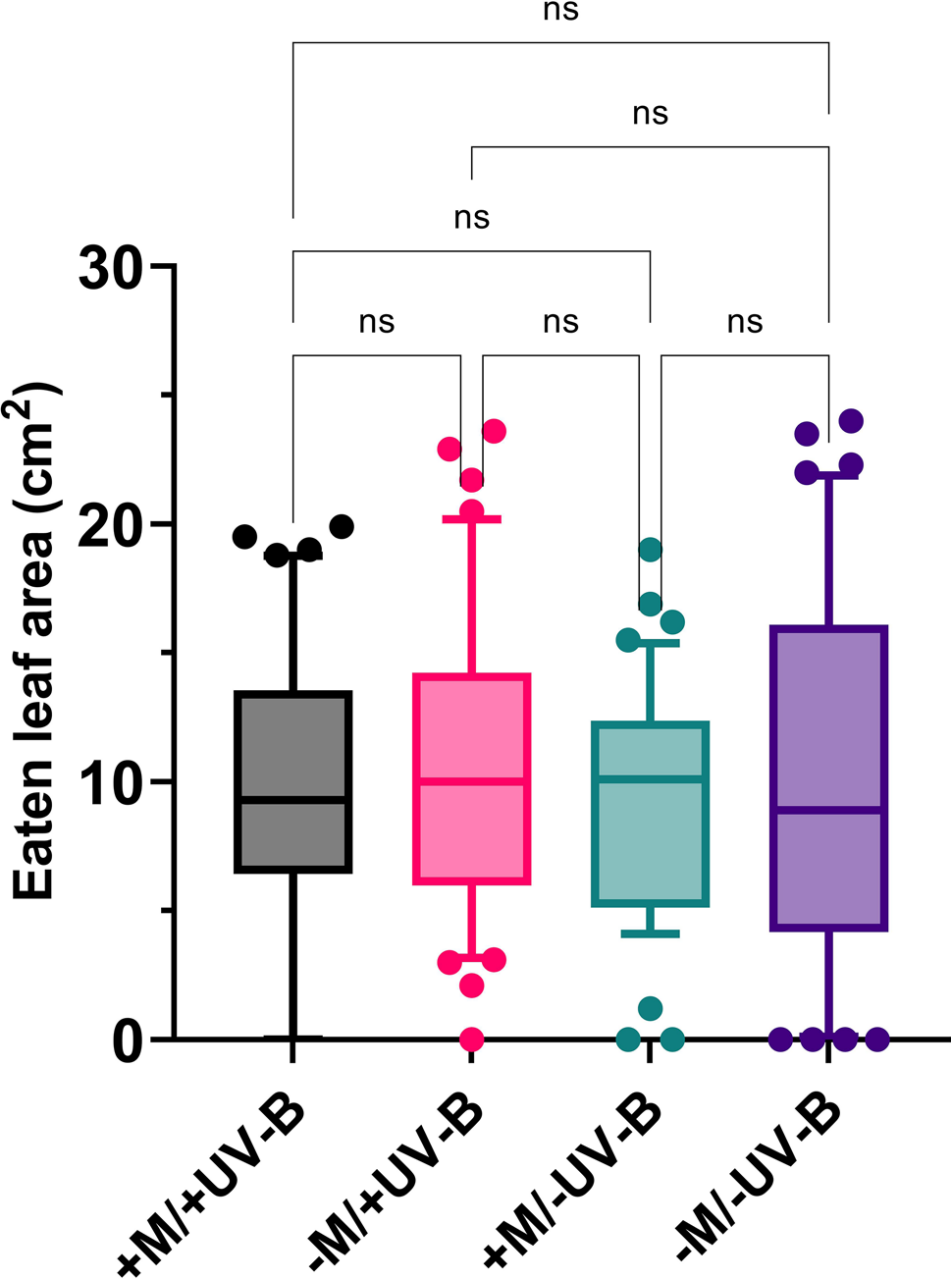
Boxplot showing the percentage of leaf area (cm^2^) consumed by larvae of *Spodoptera littoralis* in differently treated lettuce plants. + M/ + UV-B = lettuce plants exposed to UV-B radiation and inoculated with the arbuscular mycorrhizal symbiont *Funneliformis mossae*; − M/ + UV-B = UV-B-exposed plants not inoculated with *F. mossae*; + M/ − UV-B = plants inoculated with *F. mosseae* and not exposed to UV-B; − M/ − UV-B = untreated lettuce plants, unexposed and not mycorrhizal inoculated (control). Each box plot indicates the median (lower, upper quartile and extreme values, outliers); n.s., not significant (Kruskal–Wallis test, *p* > 0.05)

Since UV-B exposure increased the contents of total phenolics, flavonoids, and anthocyanins, we evaluated whether these conditions could influence the feeding preference of *S. littoralis* larvae. Caterpillars fed with treated lettuce plants (i.e. − M/ + UV-B; + M/ − UV-B; + M/ + UV-B) behave similarly to caterpillars fed with control plants (i.e. − M/ − UV-B) (Kruskal–Wallis, *χ*^2^ = 1.49, *d.f.* = 3, *p* = 0.68). In addition, the combination of both UV-B and AMF treatments did not significantly affect *S. littoralis* feeding behavior (Fig. 4). These results suggested that AMF inoculation and UV-B radiation had no indirect effects on the caterpillars.

## Discussion

### UV-B and mycorrhizal effect on lettuce plants

The potential of UV-B radiation and/or the use of AMF inocula to enhance the nutraceutical value of agricultural plants by increasing the content of health-promoting secondary metabolites has been investigated in several species, such as flaxseed (Santin et al. 2022), basil (Mosadegh et al. 2018; Nascimento et al. 2020, Battini et al. 2016), broccoli (Mewis et al. 2012; Moreira-Rodríguez et al. 2017a, 2017b), wheat (Chen et al. 2020), rice (Faseela and Puthur 2018), lettuce (Baslam et al. 2013; Avio et al. 2017), and others (Santin et al. 2021a, 2021b; Avio et al. 2018; Agnolucci et al. 2020). However, many studies are conducted using UV-B lamps as a light source (Assumpção et al. 2019; Aksakal et al. 2017; Basahi et al. 2014; Esringu et al. 2016; Lee et al. 2021; Park et al. 2007; Rajabbeigi et al. 2013; Rodriguez et al. 2014; Zhang et al. 2019), but research employing UV-B LEDs is scanty.

In this study, the lettuce plants exposed to UV-B from LED sources were 26.7% heavier and 20.8% taller than their control counterparts, suggesting a positive role of the irradiation on such biometric indexes. This observation contrasted with that of Tsormpatsidis et al. (2008), whose lettuce plants were cultivated in tunnels with or without UV (280–400 nm)-blocking filters. The authors found a lower vegetative growth (in terms of dry weight and leaf number) with the progressive increase in UV proportion. Similar to Tsormpatsidis et al. (2008), other studies reported a reduction in fresh and/or dry weight in UV-exposed lettuce (16–18 μmol m^−2^ s^−2^), although they used different lettuce cultivars (Diaz et al. 2006). UV-B irradiation negatively affected plant growth also in other plants of food interest, such as basil (18.7 kJ m^−2^ h^−1^), potato (10 kJ m^−2^ d^−1^), flaxseed 1.33 (W m^−2^), radish 7.2 (kJ m^−2^ d^−1^), rice (15.7 kJ m^−2^), barley (0.60–2.30 W m^−2^), bean (0.60–2.30 W m^−2^), and others (Chen et al. 2020; Dou et al. 2019; Santin et al. 2022; Singh et al. 2011; Teramura et al. 1991; Tevini et al. 1981). UV-B-related reduction in plant growth might be due to impairments in the cell cycle caused by DNA photo dimers (Biever et al. 2014; Yadav et al. 2020), and the likely damages to the photosynthetic apparatus and the consequent photosynthetic processes (Cuzzuol et al. 2020; Kosobryukhov et al. 2020; Piccini et al. 2020). In the current study, the increase of weight and height in the UV-B-exposed plants might be due to the lower UV-B irradiance compared to other studies, which might have lessened the appearance of UV-B-related stress-like symptoms. Indeed, in the present work, lettuce plants were exposed to low, ecologically relevant UV-B doses; therefore, it is reasonable to assume that the irradiation provided did not induce significant impairments in the plant growth and development, as conversely observed in other studies.

It is widely known from the literature that UV-B exposure can be associated with increased ROS levels, with consequent oxidative damage in plants (Gao and Zhang 2008). However, in case of moderate irradiation, plants can control oxidative stress by ROS-scavenging mechanisms that can prime plants, thus activating their pathways of defense and protecting against both abiotic and biotic stresses (Christou et al. 2022; Volkova et al. 2022). These indicate the hormetic-biphasic property of radiation and numerous chemical agents, with this dose-dependency explaining the contradictory findings between studies (Christou et al. 2022; Volkova et al. 2022). Recently, UV-B-induced oxidative stress was studied through histochemical detection of hydrogen peroxide and lipid peroxidation, as well as defense-related callose deposition in lettuce plants (Zeni et al. 2023); the authors also showed that the UV-B-induced stress was partially mitigated by the presence of AMF. Herein, the UV-B-triggered increase in the content of total phenolics, flavonoids, anthocyanins, as well as the higher antioxidant capacity, is in accordance with the results reported by Aksakal et al. (2017) and Esringu et al. (2016), who found that a 12- and 18-h UV-B irradiation (3.3 W m^−2^) effectively enhanced the contents of total phenolics and the antioxidant activity in lettuce seedlings, respectively. Similarly, a study carried out on the same lettuce cultivar (Red Salad Bowl) subjected to a 2-week UV-B radiation exposure (1 h daily, 1.69 W m^−2^) found a significantly higher flavonoid concentration. The ability of UV-B radiation to increase the content of phenolic compounds, particularly flavonoids, is mainly due to the activation of the UV-B photoreceptor UVR8, whose signaling cascade results in the upregulation of genes involved in the phenylpropanoid pathway, as observed in *Arabidopsis* (Heijde and Ulm 2012; Stracke et al. 2010) and in many other plant species (Giuntini et al. 2008; Hu et al. 2020; Santin et al. 2019, 2021b; Sheng et al. 2018; Ubi et al. 2006). The higher UV-B-triggered phenolic compounds, and consequently an enhanced antioxidant capacity, as acclimation response towards high UV-B conditions (Cloix et al. 2012; Rizzini et al. 2011) is due to the ROS-scavenging potential of such phytochemical, which can effectively prevent eventual damages to macromolecules by neutralizing the likely UV-B-induced ROS (Czégény et al. 2016; Hideg et al. 2013). In our study, we also found a significantly higher (+ 102%) concentration of total anthocyanins in the UV-B-treated plants, accompanied with a significantly redder and more homogeneous color of the leaves, as resulted from the parameters according to the CIE L*a*b* system. An enhanced content of anthocyanins was also observed by Assumpção et al. (2019), who reported a 101.35% increase of cyanidin glucoside in the UV-B-treated lettuce plants (cv. Red Salad Bowl). In line with our results, Park et al. (2007) reported that a UV-B irradiation (5-min pulse daily, 10 days totally, 0.26 kJ m^−2^ d^−1^) was effective to develop a red coloration in the leaves or red lettuce, likely due to a transcriptional activation of several flavonoid-related genes. A UV-B-induced activation of some anthocyanin biosynthetic genes was observed also with a transcriptome analysis by another study (Zhang et al. 2019), indicating that the accumulation of anthocyanin pigments in lettuce leaves occurs via transcriptional regulation through the UVR8 signaling pathway. Contrarily to our observations, Rajabbeigi et al. (2013) did not find any significant change in terms of total phenolic and anthocyanin concentration of lettuce in response to the UV-B treatment. However, the UV-B irradiation described by the authors was conducted with a much higher UV-B fluence rate (8.2 W m^−2^) and in a shorter time (5 days), indicating also a much greater dose rate; therefore, the biochemical effects triggered might be considerably different, since they are strictly dependent to both the UV-B irradiation and time of exposure (Jenkins 2017). Besides, the lettuce cultivar used (*L. sativa* var. *capitata* cv. Teodore RZ®) was different from that of our study, and physiological and biochemical responses to UV-B exposure are species- and cultivar-dependent (Rajabbeigi et al. 2013). Also, a UV-B exposure (10 h daily, 10 kJ m^−2^ d^−1^) of lettuce plants cv. Romaine was ineffective in increasing flavonoid content, although anthocyanins and total phenolics were significantly enhanced by the UV-B irradiation (Basahi et al. 2014). Photosynthetic pigments of lettuce plants were also found to be affected by the UV-B treatment in the present study. Particularly, total carotenoid concentration was increased in the UV-B-treated plants, regardless the mycorrhization, while no changes were found in the content of chlorophyll *a* + *b* or their ratio. Our findings partially agreed with the findings of Lee et al. (2021), who treated lettuce plants of New Red Fire and Two Star cultivars with 5 days of UV-B (24 h daily; 1.97 W m^−2^). The authors found no changes in chlorophyll *a* concentration in the New Red Fire cultivar, while both chlorophylls *a* and *b* were unaltered due to the irradiation in Two Star cultivar. Similarly, Li and Kubota (2009) did not find any modification in the chlorophyll content of lettuce (cv. Red Cross) plants grown under supplemental UV-B irradiation, while a significant increase in xanthophyll and *β*-carotene was detected. Consistent results were obtained also when lettuce was grown in boxes covered with either UV-B transparent or UV-B blocking films. Krizek et al. (1998) found that chlorophyll *a* and *b* contents were unchanged between lettuce plants (New Red Fire lettuce cultivar) receiving or not the UV-B solar component. Similar to our findings, Assumpção et al. (2019) reported no differences in chlorophyll and carotenoid concentration between UV-B-treated and untreated plants in the same cultivar (Red Salad Bowl) as in the present work. UV-B-induced increase of carotenoids, as reported in the present manuscript, was found also in other plant species, e.g., broccoli (Moreira-Rodríguez et al. 2017a, b), tomato (Perez et al. 2008), and canola (Qaderi et al. 2010). However, UV-B-related modulation of photosynthetic pigments is also genotype-dependent (Santin et al. 2021a; Schreiner et al. 2012). Carotenoids were found to increase by various stressors other than radiation and act as precursors of abscisic acid and some volatiles, among others, and are important for plant–insect interactions (Agathokleous 2021).

In this work, no changes in biometric and biochemical parameters were observed in mycorrhizal lettuce plants, except for a reduction in plant length. Arbuscular mycorrhizal symbiosis generally enhances plant growth; nevertheless, a reduction in plant length during symbiosis establishment may be linked to a modulation of hormonal balance during the development of fungal colonization (Liao et al. 2018). Here, colonization levels (approximately 30%) were similar to or even higher than those observed in other studies in the roots of different lettuce cultivars (Baslam and Goicoechea 2012; Avio et al. 2017). However, the sampling time (3 weeks post-inoculation), was not sufficient to detect differences in biochemical parameters between mycorrhizal and control plants. Most studies analyzing antioxidant compounds in mycorrhizal lettuce were carried out 7–8 weeks post-inoculation (Avio et al. 2017; Baslam et al. 2011a, 2011b, 2013; Goicoechea et al. 2015), when the symbiosis is generally well established with marked effects on plant secondary metabolism. Baslam et al. (2013) demonstrated that mycorrhizal inoculation increased not only lettuce growth, but also chlorophyll and/or carotenoid contents, particularly in the leaves that were most exposed to light, at such a sampling time. However, as observed in other food plants, a differential modulation of the expression patterns of genes encoding for key enzymes involved in secondary metabolite production cannot be ruled out, even 3 weeks after inoculation (Vangelisti et al. 2018). The statistically not significant results in terms of antioxidant activity, total phenolics, flavonoids, anthocyanins, and photosynthetic pigments between mycorrhizal and not-mycorrhizal lettuce plants used in the present work may be also explained by the lettuce cultivar used (Red Salad Bowl), which belongs to *L. sativa* var. *crispa*. Indeed, most data were obtained from cultivars, such as Batavia Rubia Munguía, Maravilla de Verano, Cogollos de Tudela, belonging to different botanical varieties, *capitata* or *longifolia*. For example, anthocyanin and carotenoid concentrations were usually shown to increase in the leaves of such cultivars (Baslam et al. 2011a, 2013; Goicoechea et al. 2015; Avio et al. 2018). Conversely, no effects on the enhancement of total phenolic concentration were observed in diverse investigations (Baslam et al. 2011a, 2011b, 2013), except for leaves of the cv. Batavia Rubia Munguía under optimal irrigation, in association with AMF (Baslam and Goicoechea 2012). The only study using two *L. sativa* var. *crispa* cultivars (Eluarde and Panisse) showed an increase of phenolics and antioxidant activity in the leaves of plants inoculated with different AMF isolates (Avio et al. 2017). Such data confirm that the production of antioxidant compounds is modulated by both plant genotype and AMF identity (Avio et al. 2018; Fusco et al. 2022).

### UV-B and mycorrhizal effects on caterpillar feeding behavior

Insect-plant interactions result in a wide range of outcomes depending on a variety of both abiotic and biotic factors. For instance, both UV-B treatment and AMF inoculation have been observed to stimulate plant defense mechanisms by impacting plant physiology and biochemistry (Qi et al. 2018; Zeni et al. 2023). All these changes can affect the following trophic levels, such as the interaction between treated plants and herbivore insects (Gange 2007; Qi et al. 2018). Interestingly, no treatment alone or in combination affected the food preferences of *S. littoralis* larvae*.* Concerning the UV-B treatment alone, our results are consistent with those reported by Vandenbussche et al. (2018). Indeed, *S. littoralis* caterpillars performed equally well when fed with UV-B-treated plant material as when fed with not UV-B-treated ones. This occurred despite an increase in plant antioxidant capacity due to a higher total phenolic and flavonoid contents. As reported by Qi et al. (2018) in a study on *Spodoptera litura* (Fabricius) (Lepidoptera: Noctuidae) feeding activity, the UVR8-related downstream responses are not necessarily associated with plant resistance to chewing insects. Other studies reported the marginal effect of UV-B treatment on different *Spodoptera* species (Lindroth et al. 2000; Wargent and Jordan 2013). Our findings support the hypothesis that generalist herbivores, such as lepidopteran caterpillars or polyphagous aphids [e.g., *Myzus persicae* (Sulzer)] (Zeni et al. 2023), have evolved the ability to withstand the chemical defense mechanisms of a wide range of plant species. However, it is important to consider that higher UV-B doses than the one used in this work could further enhance the biochemical defenses in terms of phenolic accumulation, without impairing plant growth and development. Therefore, deeper investigations are encouraged to study the effects of stronger UV-B irradiation on the feeding behavior of such generalist herbivores. As to the effects of AMF on *S. littoralis* feeding behavior, we can state that our results fit the analysis conducted by Heinen et al. (2018). According to their literature review, 75% of relevant papers reported no effect, and 25% reported negative effects of AMF on generalist chewing herbivores like *S. littoralis* larvae (Gange and West 1994; Vicari et al. 2002). In conclusion, the effects of UV-B exposition and AMF inoculation on foraging *S. littoralis* caterpillars may be highly dependent on the degree of specialization of the insect species. Furthermore, the responses might also depend on the plant developmental stage and on the degree of insect adaptation to variation in host plant quality due to the treatments.

## Conclusions

Overall, UV-B irradiation priming over AMF inoculation of the most suitable plant genotype may represent a promising approach to increase the nutraceutical and commercial quality of lettuce plants. The enhanced chemical defenses of plants did not impact the feeding behavior of *S. littoralis* larvae, probably due to (i) the generalist feeding traits of such herbivores, (ii) the mild UV-B treatments, and (iii) the limited time for the induction of mycorrhizal effects on plant secondary metabolism (see also Zeni et al. 2023). Therefore, further investigations are needed to assess whether different doses of UV-B/AMF and exposure durations (smaller or greater) that increase plant antioxidant defenses within a hormetic framework may alter the feeding behavior of oligophagous insect pests.

## Data Availability

Data are available from the corresponding author at reasonable request.
